# Multiple Sclerosis: LIFNano-CD4 for Trojan Horse Delivery of the Neuro-Protective Biologic “LIF” Into the Brain: Preclinical Proof of Concept

**DOI:** 10.3389/fmedt.2021.640569

**Published:** 2021-04-07

**Authors:** Raul de la Flor, Janette Robertson, Rostislav V. Shevchenko, Mo Alavijeh, Sean Bickerton, Tarek Fahmy, Su M. Metcalfe

**Affiliations:** ^1^Pharmidex Pharmaceutical Services Ltd, Atlas House, London, United Kingdom; ^2^Yale School of Engineering and Applied Science, New Haven, CT, United States; ^3^University of Cambridge Clinical School, LIFNanoRx Ltd, Cambridge, United Kingdom

**Keywords:** LIF, BBB, CD4^+^ T lymphocyte, PLGA nanoparticles, pK/pD, EAE, multiple sclerosis

## Abstract

Multiple sclerosis (MS) is a demyelinating autoimmune disease that attacks the brain, with year-on-year loss of brain volume, starting late teens and becoming manifest late twenties. There is no cure, and current therapies are immunosuppressive only. LIF is a vital stem cell growth factor active throughout life—and essential for health of the central nervous system (CNS), being tolerogenic, myelinogenic, and neuroprotective. Nano-formulation of LIF (LIFNano) using FDA-approved PLGA captures LIF's compound therapeutic properties, increasing potency 1,000-fold when targeted to CD4 (LIFNano-CD4). Moreover, circulating CD4^+^ lymphocytes are themselves regulated by LIF to express the Treg phenotype, known to release T cell-derived LIF upon engagement with cognate antigen, perpetuating antigen-specific self-tolerance. With the longer-term aim of treating inflammatory lesions of MS, we asked, does LIFNano-CD4 cross the blood–brain barrier (BBB)? We measure pK and pD using novel methodologies, demonstrate crossing of the BBB, show LIF-cargo-specific anti-inflammatory efficacy in the frontal cortex of the brain, and show safety of intravenous delivery of LIFNano-CD4 at doses known to provide efficacious concentrations of LIF cargo behind the BBB.

## Introduction

There are no effective treatments of central nervous system (CNS) neurodegenerative diseases, where one block to progress is effective systemic delivery of a therapeutic that crosses the blood–brain barrier (BBB). MS affects 2.3 million people worldwide, often starts in young adulthood, and is often progressively disabling, costing the economy an estimated 3 billion US dollars each year. MS pathology consists of inflammatory demyelination of CNS axons causing neurological symptoms initially, when efficient nerve impulse conductance becomes compromised: later, nerve death occurs due to the loss of glial-derived metabolic support to the axon, leading to irreversible neurodegeneration.

Having identified LIF as a natural regulator of neuro-immune health, we developed LIFNano and demonstrated that (i) LIFNano-CD4 promotes antigen-specific Treg and suppresses TH17 immunity *in vivo* (1); (ii) LIFNano-CD4 promotes recovery from EAE in a stringent Biozzi mouse model of neuroprotection (2); (iii) LIFNano-NG2 is a highly potent inducer of myelin repair *in vivo* (3)—far superior to other myelinogenic agents; and (iv) LIFNano-CD4 partially reverses paralysis in the Hooke EAE model of progressive MS (manuscript in preparation). These properties are likely to extend to man, since both human and non-human primate studies (1) confirm efficacy of LIFNano-CD4 *ex vivo*.

Here, we asked, does LIFNano-CD4 cross the BBB following intravenous delivery? Novel methods to measure LIF cargo behind the BBB were developed, and delivery into the brain parenchyma was confirmed, as also was efficacy in two models of EAE (RRMS and PMS), and safety in a formal preclinical trial.

## Methods and Results

### Do PLGA Nanoparticles Targeted to CD4 Cross the BBB?

**Figure 1:**
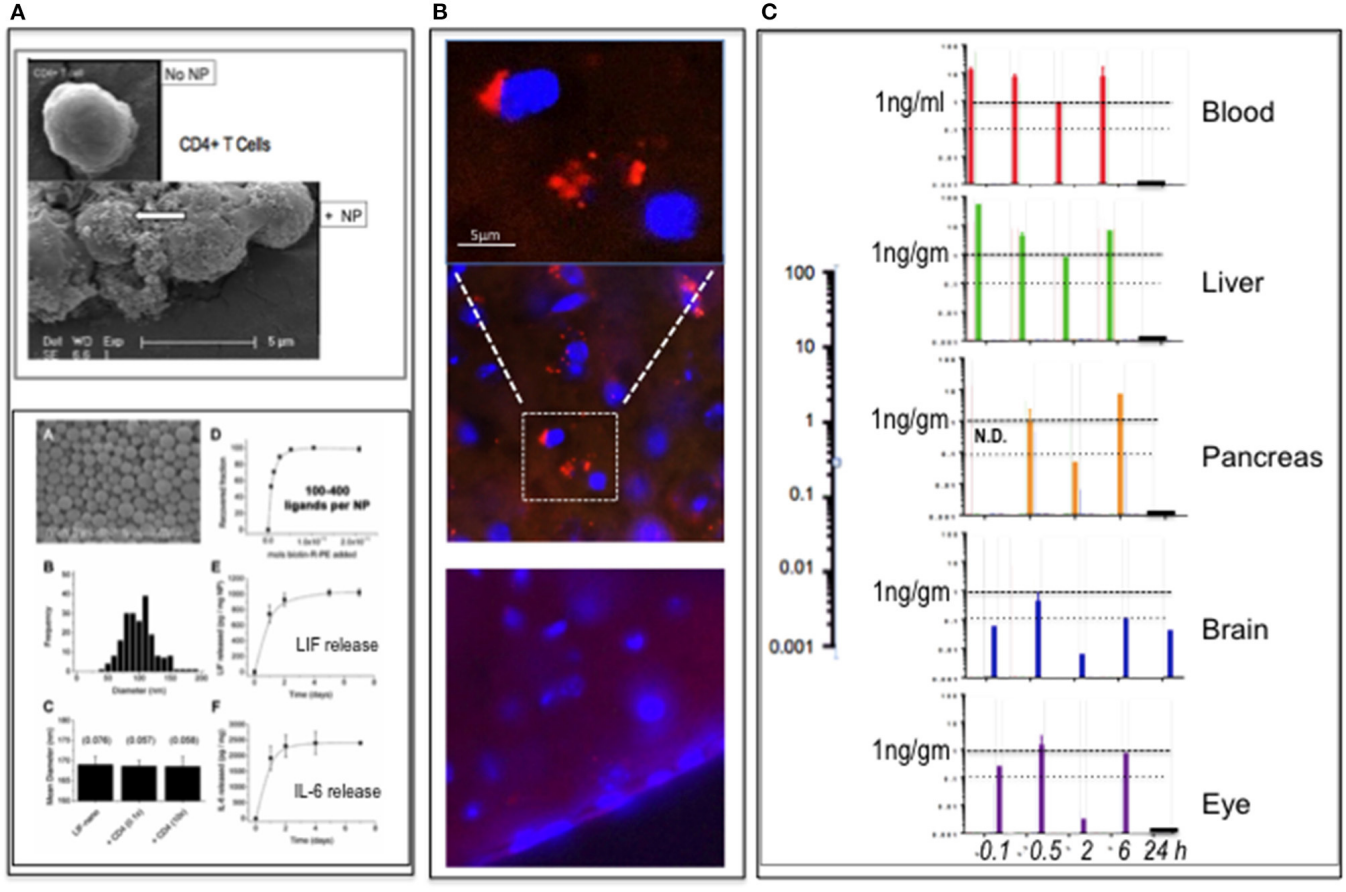
PLGA-nanoparticles targeted to CD4. **(A)** Top panel: PLGA nanoparticle (NP) targeted to CD4 binding to CD4^+^ T cells (arrow). (SEM) Bottom panel: modified from Park et al. (1), showing PLGA nanoparticles (A); size distribution (B); constant diameter of functionalized particles (C); number anti-CD4 ligands per particle (D); release rate LIF cargo (E); and release rate IL-6 cargo (F). Full details in Park et al. (1). **(B)** Detection of rhodamine B (rhoB) cargo in PLGA-CD4 NP in brain parenchyma 6 h following i.v. delivery to mouse tail vein using IHC. The upper panel is magnified and image enhanced to reduce the background compared to the middle panel of the 6-h time-point. At 24 h (lower panel), there was no definitive evidence of rhoB using IHC. **(C)** pK and pD of RhoB-PLGA-CD4. Detection of rhoB cargo using HPLC-FD fluorescence quantification of blood, liver, pancreas, brain, and eye (retina) tissues at 0.1, 0.5, 2, 6, and 24 h. The 0.1-h sample of the pancreas was not done. Abscissa values are log-scale (expanded on left): a horizontal line marking the 1-ng/gm per tissue is provided for ease of comparison.

Using PLGA-CD4 nanoparticles, illustrated in Figure 1A with physical properties of cargo release and particle binding to target cells (1), we first sought to visualize the particles *in vivo* following intravenous (i.v.) delivery and to also determine pK and pD by adding the dye rhodamine B to the cargo (Rho-PLGA-CD4). Rhodamine B was selected as a validated method where free rhodamine B is non-toxic and excluded from the brain parenchyma (4). Histological verification of BBB crossing by nanoparticles is a valid method to assess BBB properties of nanoparticles and distribution within the brain (5). Here, to obtain an estimation of the brain pharmacokinetics of the nanoparticle cargo within the nanoparticles after systemic administration, we combined histological verification with HPLC-FD fluorescence quantification of rhodamine B at various time points. Methods are detailed in the Supplementary Material.

C57BL/6 mice (*n* = 15) were assigned to four groups (*n* = 1 per group per time-point; 5 time-points) and dosed i.v. into the tail vein with either (i) unlabeled PLGA-CD4 nanoparticles (groups 1 and 2) or (ii) Rho-PLGA-CD4 (groups 3 and 4). Nanoparticle (NP) dose was 30 mg NP/kg mouse weight in 0.9% saline. Mice were culled and tissues collected at 5, 30 min, 2, 6, and 24 h as described in the legend to Figure 1. Tissues collected were brain, eyes, liver, pancreas, and plasma. Group assignments were Groups 1 and 3 for HPLC-FD; Groups 2 and 4 for histology.

The results summarized in Figure 1B show histological detection of rhodamine B nanoparticles in the brain at 6 h post-i.v. administration. Figure 1C shows HPLC-FD detection of rhodamine B in all tissues collected up to 6 h: at 24 h, only the brain remained positive. In a repeat experiment using i.v. delivery, the findings were similar: here spleen was also assessed: spleen showed low rhodamine per mg at 30 min relative to blood, liver, pancreas eye, and brain. This suggests that the CD4-targeted nanoparticles—known to bind CD4^+^ cells *in vitro* (Figure 1A)—became bound to circulating CD4+ve lymphocytes with rapid kinetics *in vivo*. This is in accord with Schmid et al. (6), who, using CD8-targeted PLGA nanoparticles, observed >90% binding to CD8^+^ cells within 1 h of i.v. delivery in mouse.

### Do hLIF-Loaded PLGA-CD4 Nanoparticles Cross the BBB?

Having confirmed crossing of the BBB by carrier nanoparticles, we next sought to confirm delivery of LIF as cargo into the brain. Given the background of mouse LIF, we used human LIF-loaded nanoparticles, designated LIFNano-CD4, to discriminate the cargo-derived LIF delivery. It is important to note that here we are detecting released hLIF that had been carried as cargo inside the PLGA nanoparticles, these in turn being attached to CD4 T cells. The cells act as Trojan Horse-type vehicles, and the particles gradually dissolve releasing their LIF cargo. Only this released hLIF becomes detectable to the ELISA, as distinct from the previous question that addressed the nanoparticles *per se*—made detectable by rhodamine labeling.

Human LIF-specific ELISA methodology was developed for quantifying rhLIF in mouse brain matrix. This concluded that (i) extraction of LIF from brain parenchyma tolerates up to 10% DMSO. (ii) Without DMSO extraction, there is ~25% degradation of LIF-PLGA-CD4 nanoparticles over 24 h at room temperature: this is comparable to the rate previously reported (1). (iii) Incubation of the brain matrix homogenized in PBS for 24 h at RT gave the most reliable results. (iv) Addition of 10% DMSO accelerated LIF release, but this was incomplete at 6 h incubation at RT (also see Supplementary Methods).

**Figure 2:**
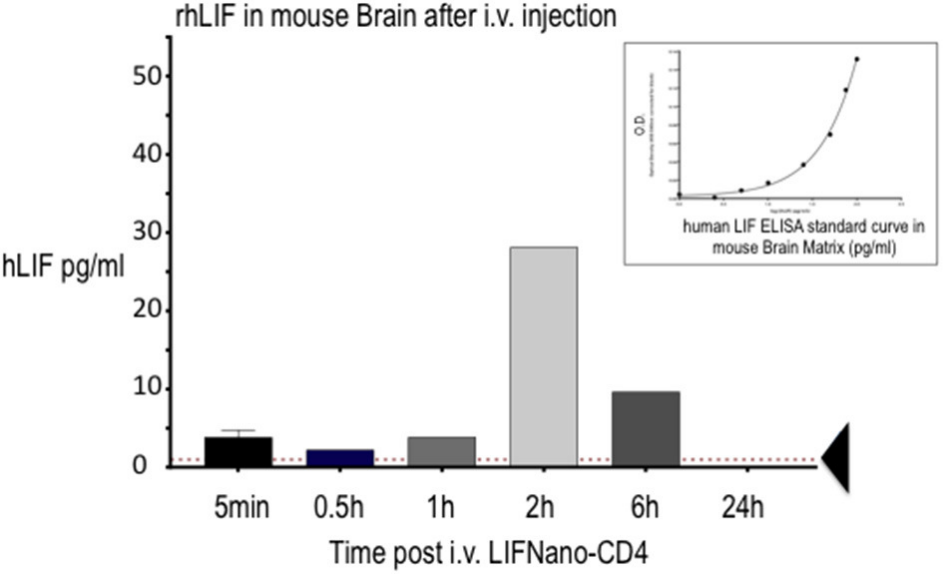
Levels of rhLIF measured in the mouse brain following delivery of hLIFNano-CD4 to the mouse tail vein. Individual mice were culled at the indicated times and brain cryopreserved until analysis. Extracted samples were measured for hLIF levels by human-LIF-specific ELISA using a hLIF standard curve prepared in the mouse brain matrix (insert): note the effect of the matrix on the optical density (O.D.) readout contrasting to the standard linear readout when measured in diluent alone. Threshold of ELISA sensitivity is indicated by the arrow.

Figure 2 shows measurement of hLIF in mouse brain parenchyma after i.v. injection of LIFNano-CD4. Levels peaked at 2 h (28 pg/ml homogenate) decreasing to 12 pg/ml by 6 h. We concluded that hLIF cargo reached the brain parenchyma—crossing the BBB—when formulated as LIFNano-CD4 and dosed i.v. The peak at 2 h may relate to early burst of released cargo evident in Figure 1A. The continued paracrine-type release of cargo evident at 6 h indicates a sustained exposure to LIF as the particles slowly biodegrade to carbon dioxide and water. Once released as cargo, the soluble LIF has a half-life of some 20 min.

### Is LIF Delivered by LIFNano-CD4 Nanoparticles Efficacious Behind the BBB?

Having confirmed that PLGA-CD4 nanoparticles cross the BBB (Figure 1) and that the biologic LIF as cargo (LIFNano-CD4) also crosses the BBB, retaining biological activity as determined by ELISA (Figure 2), we next sought to confirm the efficacy of LIF following Trojan Horse-type delivery via CD4^+^ T lymphocytes. Here LIFNano-CD4 was loaded with mouse LIF: mouse LIF, but not human LIF, is bioactive in the mouse. Two *in vivo* models of MS were used: the Hooke model following a progressive course of disease, and the ABH (Biozzi) model following a relapsing, remitting disease course.

HOOKE MODEL (PMS): In the Hooke model of progressive experimental allergic encephalitis (EAE), EAE is induced by active s.c. immunization in C57Bl/6 mice using MOG 35–55 peptide/CFA emulsion day 0: then at 2 h, and again at 26 h, 150 ng pertussis toxin i.p. is given. Following immunization, paralysis is progressive with full paralysis of tail and hind limbs by day 14: experiments were started on day 15. There were three groups: (i) untreated controls—no EAE; (ii) established EAE mice treated with “empty” PLGA-CD4 nanoparticles (i.e., no cargo); and (iii) established EAE mice treated with LIFNano-CD4 nanoparticles. Treatment was daily 1 mg/dose i.p. starting at day 15: the i.p. route was used to avoid stress of i.v. delivery. Mice were culled at 25 d post-initiation of EAE induction. Sample collection included blood and brain: cytokine levels were measured in both plasma (blood compartment) and the brain's frontal cortex (CNS compartment).

**Figure 3:**
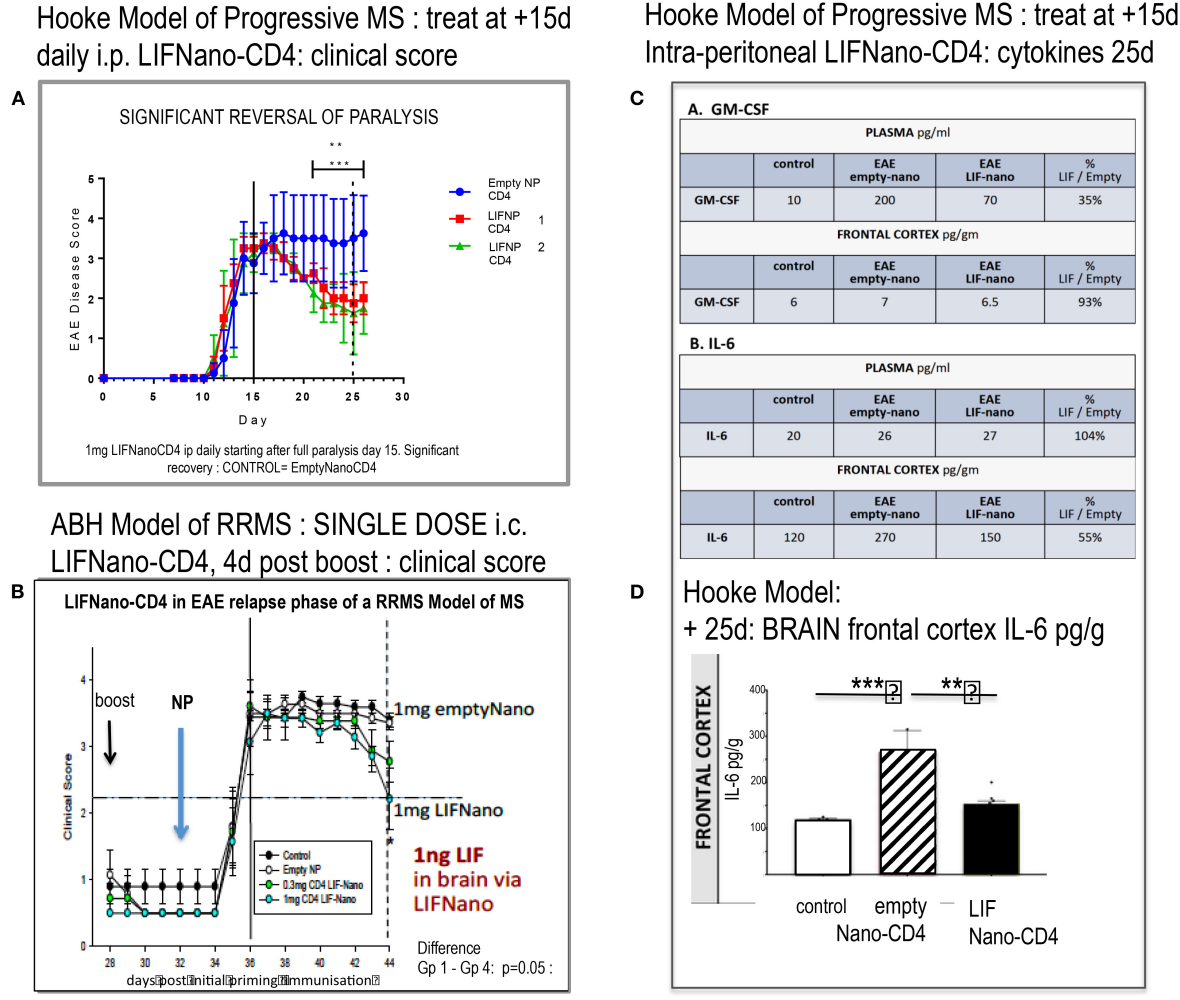
Effect of LIFNano-CD4 in two mouse models of EAE. **(A)** The Hooke model of progressive MS as described in the text and **(B)** the Biozzi model of relapsing–remitting MS (RRMS) used to test for neuro-protective efficacy as detailed in Al-Izki et al. (7). Briefly, Biozzi ABH are immunized using spinal cord homogenate plus adjuvant to induce first relapse measured by clinical score. Following spontaneous recovery, primed mice receive a boost immunization (28 d) followed by LIFNano-CD4 (32 d) prior to onset of second relapse. Clinical scores were continued out to 44 d. **(C)** Mice from **(A)** were culled and cytokines measured (see Supplementary Material). The table **(C)** shows GM-CSF and IL-6 cytokine responses in plasma and brain at 25 d, where treatment was given daily 15–24 d as detailed in the text. **(D)** Histogram of IL-6 levels in the brain frontal cortex of controls (open), EAE (Hooke) treated with empty Nano-CD4 (diagonal), and EAE (Hooke) treated with LIFNano-CD4 (black). Statistical Significance *** > 0.001; ** > 0.01. Full multiplex data is provided in Supplementary Material.

Figure 3A shows that LIFNano-CD4 treatment resulted in significant reversal of paralysis in the Hooke model. This was evident in two independent groups (1 and 2) receiving LIFNano-CD4, vs. the matched control group treated identically but with Empty-Nano-CD4 (i.e., no LIF cargo) where continuous disease occurred.

Notably, at the start of treatment, EAE disease was fully established with clinical score 3.5. Given that this Hooke model is progressive, the efficacy and speed of LIFNano-CD4 in partially reversing paralysis starting as early as 4 days was unexpected. However, LIFNano-NG2 induces maturation of oligodendrocyte precursor cells to myelin-competent oligodendrocytes within 3 d and promotes high-quality myelin repair *in vivo* (3). In the current work, we target CD4, also shown to be neuro-protective (see later). LIF switches CD4 cells away from myelin-directed TH17 toward Treg, able to support myelin repair and neuro-protection given that Treg releases endogenous LIF (8).

In the second EAE MODEL (RRMS), we asked, what therapeutic dose of LIF is required in the CNS to be neuro-protective in relapsing disease? Using the Biozzi mouse model of relapsing remitting MS, four groups of 12 animals each were used to test a single intra-cranial injection of PLGA nanoparticles targeted to CD4: Group 1 = untreated control; Group 2 = empty-Nano-CD4; Group 3 = LIFNano-CD4 0.3 mg/mouse; Group 4 = LIFNano-CD4 1.0 mg/mouse. Treatment was immediately prior to an induced relapse. The key findings (Figure 3B) were (i) no effect on rate of relapsing paralysis: thus, the induced peripheral anti-myelin immune response was not affected by the LIFNano-CD4 delivered direct to the brain. (ii) A significant dose-dependent effect on rate of recovery from the induced relapse that was dependent on the LIF cargo increased the recovery rate which is a hallmark of neuro-protection. Based on LIF load, it was calculated that a single dose of 1 ng LIF i.c. in the mouse brain sufficed to at least partially protect neurons from pathogenic inflammation.

Having shown *in vivo* efficacy, we next looked at *in vivo* cytokine behavior linked to LIF when delivered by LIFNano-CD4 as described in Supplementary Methods and Results. The experimental mice from the Hooke Model reported in Figure 3A were analyzed, comparing samples from the blood and the brain compartments. Focusing on GM-CSF and IL-6, an unexpected differential behavior was discovered. For GM-CSF in plasma, levels became elevated from 10 pg/ml (control) to 200 pg/ml in the EAE mice treated with empty nano-CD4 particles. In marked contrast, in those mice receiving LIFNano-CD4, plasma GM-CSF was significantly reduced, at 70 pg/ml. The profound effect of EAE on induction of GM-CSF was limited to the periphery, since GM-CSF levels in the brain frontal cortex parenchyma were the same in all groups (6–7 pg/ml). Thus, an effect of LIF (derived from i.v. LIFNano-CD4) was confirmed, and this effect was selective for the periphery at the time point measured. The same samples were assayed for IL-6. Here, in marked contrast to GM-CSF, in the plasma IL-6 levels were constant (20–27 pg/ml) in the three treatment groups. However, there was a striking difference in the brain. Here IL-6 was significantly increased in EAE treated with empty-PLGA-CD4 (170 pg/ml control vs. 270 pg/ml EAE/empty PLGA-CD4: *p* = 0.001). In contrast, EAE mice receiving LIFNano were equivalent to control levels (150 pg/ml; *p* = 0.01). Thus, for IL-6, an effect of LIF derived from LIFNano-CD4 was confirmed, and this effect was selective for the brain (Figures 3C,D).

We conclude that LIF is efficacious behind the BBB when delivered to the periphery as LIFNano-CD4. The differential behavior between GM-CSF and IL-6 in plasma vs. brain within the same samples further confirmed efficacy, wherein each marker was an internal control for the other. The findings also provide evidence for the integrity of the BBB at the time of sampling. Of all the cytokines measured, in the CNS compartment only IL-6 showed significant differences in concentration correlating with therapy (Supplementary Material).

Taken together, the two independent EAE models converge as proof of LIFNano-CD4 being neuro-protective and anti-inflammatory behind the BBB including when delivered in the periphery.

### Tolerability of LIFNano-CD4

Finally, we asked, is LIFNano-CD4 safe? Here a single ascending dose (SAD) preclinical trial in mice followed the protocol outlined in Table 1. For regulatory approval, the therapeutic is required to be that proposed for use in man. Accordingly, LIFNano-CD4 particles were loaded with rhLIF.

**Table 1:** hLIFNano-CD4: Single Ascending Dose Preclinical Toxicity Trial: Mouse.

**GROUP**	**1**	**2**	**3**	**4**
Test Item	PBS control vehicle	hLIFNano-CD4	hLIFNano-CD4	hLIFNano-CD4
Concentration	0mg/kg	50mg/kg	100mg/kg	150mg/kg
Mouse Male	3	3	3	3
Mouse Female	3	3	3	3

***Experimental observations and measurements:** Mortality, clinical observations up to 7 days, post-dose observations (0.5, 1, 2, 4, 24h), body weight (7d), clinical toxicity assessment, haematology, clinical chemistry (at day 7). Blood and organs collected for analysis and histology*.

***Main conclusions:** LIFNano-CD4 is tolerated at 50mg/kg. Mild-moderate transient toxicity observed at 30-60 min post iv administration at 100 and 150 mg/kg, which resolved at 2h. Haematology and clinical chemistry parameters all within normal range, but some parameters elevated in a dose-dependent manner which may indicate potential toxicity at higher doses*.

Briefly, four groups of six CD-1 mice (three male plus three female at 6 weeks of age) were treated with a single i.v. dose of LIFNano-CD4, or vehicle control. LIFNano-CD4 was given at three concentrations: 50, 100, and 150 mg/kg. Daily monitoring was to day 7, when mice were culled for assessments of clinical toxicity, hematology, and clinical chemistry. The data showed safety at 50 mg/kg LIFNano-CD4, with transient mild effects at 100 mg/kg and 150 mg/kg. It was calculated from the previous pK data that 50 mg/kg LIFNano-CD4 i.v. will deliver some 250 pg LIFNano-CD4 behind the BBB. This dose is on a par to that shown to be neuro-protective in the Biozzi mouse EAE model of relapsing MS via intra-cranial delivery. The design of the preclinical toxicology is shown in Table 1 with conclusive findings in the Supplementary Material.

In summary, we deduced that i.v. LIFNano-CD4 is a safe and effective way to treat inflammatory disease behind the BBB. Notably, the downregulation of pathogenic levels of IL-6 in the CNS compartment by LIFNano-CD4 is the first report exploiting peripheral CD4^+^ T lymphocytes to deliver LIF behind the BBB using nanotechnology and an FDA-approved gel.

## Discussion

LIF is a vital neuro-protective growth factor required for brain health throughout life. We designed LIFNano-CD4 to harness these natural properties of LIF, exploiting CD4^+^ T lymphocytes for Trojan Horse-type delivery to sites of inflammation *in vivo*. We show for the first time that LIFNano-CD4 (i) cross the BBB; (ii) reduce pathogenic levels of IL-6 that accumulate behind the BBB during demyelinating autoimmunity; and (iii) are non-toxic at efficacious i.v. doses in a formal preclinical SAD trial.

In addition, we identify (i) a method to quantify LIF in the brain parenchyma and (ii) a method to differentiate between PLGA-LIF and soluble LIF derived from PLGA-LIF nanoparticles: this allows temporal differential pK analyses. This preliminary report identifies the option of further experiments where pharmacokinetics will be repeated in the EAE models wherein LIFNano-CD4 is given i.p with the time course corresponding to times of treatment.

Our unexpected discovery that LIF decreases pathogenic levels of inflammatory IL-6 behind the BBB is of profound importance. It is known that both LIF and IL-6 are naturally transported across the BBB via receptor-mediated transcytosis (9): also that, in the BBB, IL-6 suppresses gp190, the LIF receptor that acts as the LIF-transporter (10). In T lymphocytes, IL-6 directly inhibits gp190 transcription, biasing lineage maturation toward TH17 immunity (11), TH17 being an initiating driver of multiple sclerosis in human and EAE in animal models (12). A counter-regulatory binary switch exists between LIF and IL-6, mechanistically operating by competition for gp130, the signaling receptor subunit for both LIF and IL-6 (8). LIF signals through the gp130/gp190 hetero-dimeric receptor. IL-6 signals through the gp130/gp130 homo-dimeric receptor. By shutting down gp190 transcription, IL-6 effectively commandeers use of gp130 in the plasma membrane, tipping the balance toward pathogenic inflammation and away from neuro-protective tolerogenesis linked to Treg cell immunity (8). In addition to MS with lesions linked to TH17 activity against myelin, the age-related dementias are causally linked to increasing levels of circulating IL-6 (13, 14). High IL-6 will reduce transport of endogenous LIF across the BBB, again tipping the balance toward inflammation within the CNS [discussed in (15)]. LIFNano-CD4 is the first identified means to directly intercept IL-6-mediated pathogenesis in the CNS. By acting to reset the natural balance of the LIF/IL-6 axis, progressive CNS disease may be halted or even partially reversed using LIFNano-CD4.

Other studies have utilized different means to exploit LIF including adenoviral constructs of LIF or LIF-related cytokines: for example, to demonstrate myelin repair (16). In patients with MS, Janssens et al. (17) found a correlation with IL-6 that could be shifted *ex vivo* using LIF to tip the immune balance toward regulatory T cells. More recently, bone marrow stem cells have been used to target a therapeutic LIF transgene to muscle via the immune system, ameliorating muscular dystrophy in a mouse model (18). Others have explored Trojan-Horse delivery of nanoparticle-encapsulated drugs via T cells in mice (6): our use of biologics to generate targeted biomimetics is novel and drug-free: it harnesses T cell biology concurrent with delivering growth factor by paracrine delivery achieving 1,000-fold potency without toxicity (19).

The accumulating data identifies LIF as a powerful therapeutic growth factor. Use of nanotechnology combined with deep biology of fundamental lineage control of the CD4^+^ T lymphocyte has enabled a new generation of biomimetics that operate as “intelligent” immune-based therapeutics. Unlike bi-specific antibodies, or antibody–drug conjugates, biomimetics provide a highly potent means of cooperative synergy harnessing the immune system to reset natural homeostasis on both sides of the BBB. Once reset, endogenous pathways sustain a therapeutic value in the absence of the transient biodegradable mimetic.

## Conclusion

Using FDA-approved PLGA gel nanoparticles, the neuro-protective tolerogenic properties of LIF can be delivered as cargo across the BBB by attachment to CD4^+^ T lymphocytes. Unlike pK and pD of drugs, attachment to circulating immune cells focuses the host's tissue exposure to cargo to the targeted cell, while the biodegradable nanoparticle exists transiently—released LIF is degraded within minutes and the PLGA carrier degrades to carbon dioxide and water over some 6 days. Efficacious levels in the CNS are reached following i.v. delivery, with no toxic side effects.

We conclude that Trojan Horse delivery of the biologic “LIF” into the brain using LIF-loaded PLGA nanoparticles functionalized with anti-CD4 is a validated therapeutic candidate to treat multiple sclerosis.

## Data Availability Statement

The raw data supporting the conclusions of this article will be made available by the authors, without undue reservation.

## Ethics Statement

The animal study was reviewed and approved by RF, Pharmidex Ltd.

## Author Contributions

RF, JR, RS, and MA: pK pD and tissue (EAE) analyses and toxicology. SB and TF: Hooke EAE model and LIFNano preparation. RF: development of novel assays to distinguish free cargo and nano-encapsulated cargo. SM: conceptualization, supervision, funding acquisition, writing, review, and editing. All authors contributed to the article and approved the submitted version.

## Conflict of Interest

SM is Founder and CEO of LIFNanoRx Ltd., and is recipient of I-UK BioMedical Catalyst Biomedical Catalyst Award Early 2016 (Grant Number 510136) that has funded the work reported here in toto: RF, RS, and MA were employed by Pharmidex Ltd a contract research organization who were partner on the Grant 510136 with specific brief to undertake the pK, pD, and toxicology reported here. TF and SB of Yale University were funded by the grant 510136 to perform the EAE experiments. All the authors declare that the research was conducted in the absence of any commercial or financial relationships that could be construed as a potential conflict of interest.
