# Supplementary material for: Multiple Sclerosis: LIFNano-CD4 for Trojan Horse Delivery of the Neuro-Protective Biologic “LIF” Into the Brain: Preclinical Proof of Concept
Source: Front Med Technol. 2021 Apr 7;3:640569. doi: 10.3389/fmedt.2021.640569 (PMC8757767; doi:10.3389/fmedt.2021.640569)
Supplement: Supplementary file 1 [file Data_Sheet_1.docx]

Biomimetic LIFNano-CD4 for MS

SUPPLEMENTARY

METHODS

*Materials*

LIFNano, PLGA nanoparticles and PLGA nanoparticles labelled with Rhodamine B were obtained from Yale. Nanoparticles were stored at -80℃ and coated with anti-CD 4 antibody (Anti-Mouse CD4 Biotin Clone RM4-4, eBioscience) on the day of the experiment. Rhodamine B, to prepare standard calibration curves in plasma and tissue, was purchased from Sigma.

*Anti-CD coated PLGA nanoparticles formulation:*

PLGA nanoparticles were resuspended in 0.5 ml PBS under sterile conditions. Then, 0.5 ml of sterile biotinylated anti-CD4* (0.5mg/ml) was added to the suspension and incubated at RT for 30 min with occasional mixing. Anti-CD4 coated nanoparticle suspension was centrifuged, and nanoparticle pellet was resuspended again in 1ml of sterile PBS. The suspension was centrifuged, and the resulting pellet resuspended in sterile PBS to yield the desired concentration. The suspension was used immediately after formulation and precipitation was avoided during dosing by gentle shaking.

*Mice*

All studies were performed at an Association for Assessment and Accreditation Laboratory Animal Care International (AAALAC) accredited establishment and under protocols approved by Home Office license according to Animals (Scientific Procedures) Act 1986. Mice were given ad lib food and water and were kept in a 12/12-hour light dark cycle.

*Anti-CD4 coated PLGA particles pharmacokinetics*

BALB/C mice were dosed intravenously with either blank PLGA nanoparticles or PLGA nanoparticles labelled with rhodamine B at 15 mg/Kg in PBS. Mice were anaesthetized and blood collected by cardiac puncture at 5 min, 30 min, 2h, 6h and 24h after i.v. administration. Then, animals were perfused with warm PBS. Brains were dissected and snap frozen. Samples were kept at -80°C until determination of rhodamine by HPLC-FD. For histology, animals were perfused with 10 ml of warm PBS followed by 10 ml of 4% PFA. Brain, eyes, pancreas, spleen, and liver were dissected and transferred to appropriate vials containing 4% PFA. Tissue was fixed in PFA, cryoprotected with sucrose and embedded in OCT.

*Rhodamine analysis by HPLC-FD*

Plasma samples were prepared for analysis by protein precipitation with 75% acetonitrile, centrifugation, filtration of supernatant and transfer to 0.2 ml vials. Liver, brain, eye, spleen, and pancreas samples were homogenized (1:3, w:v) in 75% acetonitrile and then prepared for analysis in the same way as for the plasma samples. Calibration standards consisted of control plasma spiked with analyte over the range 0.01-10 ng/ml, and diluted control brain, liver, spleen, and pancreas homogenate spiked with analyte over the range 0.01-10 ng/g. These were prepared and analysed in the same way as the samples. Rhodamine analysis was performed by HPLC-FD, using a Jasco HPLC ternary system coupled to a Jasco FP-2020 fluorescent lamp (excitation 550 nm, emission 580 nm). Gradient elution was employed for the separation of rhodamine, using acetonitrile and 100 mM ammonium acetate, both acidified with 0.1% formic acid, and a C-18 column (ACE 5 C18, 5 μm, 150 X 3 mm). Chromatographic data was processed using Jasco ChromNav software.

*LIFNano pharmacokinetics*

BALB/C mice were dosed intravenously with either anti-CD4 coated blank PLGA nanoparticles or LIFNano (45 mg/Kg, i.v.). Animals were anaesthetised and blood collected by cardiac puncture at 5 min, 30 min, 2h, 6h and 24h after i.v. administration. The blood was collected into pre-prepared, labelled polypropylene tubes containing 6µl of heparin. The blood samples were centrifuged at 21000g, 4°C for 5 minutes and the resulting plasma transferred into pre-labelled polypropylene tubes. Immediately after blood sampling, the animals were perfused with warm PBS to remove residual blood and brains were dissected and snap frozen. Samples were kept at -80°C until determination of LIF by ELISA.

*hLIF quantification in plasma and brain by ELISA*

hLIF in plasma was quantified using Human LIF Quantikine ELISA Kit (R&D Systems) following manufacturer instructions. hLIF in brain was quantified using Human LIF Quantikine ELISA Kit (R&D Systems) with the following modifications: brains were homogenised in PBS (1:4 w:v) using a bullet blender and homogenates were put through two freeze-thaw cycles before plating alongside matrix matching calibration standards (5-100 pg/ml). Plate was incubated for 24h at 37℃ and manufacturer instructions were followed in subsequent steps.

*Cytokine determination by Luminex magnetic bead assay*

Brain and plasma cytokine levels from control, EAE vehicle and EAE LIFNano treated groups were measured using a Luminex cytokine assay kit (R&D Systems), following the manufacturer’s instructions. The following cytokines were determined in a single assay: CCL20, GM-CSF, IFNγ, IL-10, IL-12p70, IL-13, IL-17/IL-17A, IL-17E/IL-25, IL-1β, IL-27, IL-4, IL-5, IL-6 and TNFα.

*Maximum tolerated dose study*

6 weeks old male and female CD-1 mice were sourced from a UK-approved designated animal breeding facility at Charles River (UK) Limited. The health status of the animals was evaluated in accordance with accepted veterinary practice and animals were confirmed to be healthy and suitable for a study. An approved rodent diet and mains tap water (in bottles) were available *ad libitum* during the acclimatisation period and entire duration of the study. Approved cages, bedding and environmental enrichment were used for the study. The CD-1 mouse is a suitable rodent species for toxicity testing, acceptable to regulatory authorities and for which extensive background data were available. Animals were administered a single dose intravenously via lateral tail vein (0, 50, 100 and 150 mg/ml, 7.5 ml/kg) followed by detailed observations at the defined time-points during the next 7 days.

Based on clinical observations, body weight and clinical pathology data, there is evidence to suggest that hrLIFNano LN-001 is tolerated at 50 mg/kg, but causes mild to moderate transient toxicity up to 60 minutes post-dose at 100 and 150 mg/kg, although all animals fully recover by 2 hours post-dose and the compound is well tolerated during remaining 7 day test period.

*MOVIES*

*(A) HOOKE Model EAE: mice at day 19: Untreated*

*(B) HOOKE Model EAE : mice at day 19: Treated with LIFNano-CD4 i.p. day15 - day18*
