# Supplementary material for: Multiple Sclerosis: LIFNano-CD4 for Trojan Horse Delivery of the Neuro-Protective Biologic “LIF” Into the Brain: Preclinical Proof of Concept
Source: Front Med Technol. 2021 Apr 7;3:640569. doi: 10.3389/fmedt.2021.640569 (PMC8757767; doi:10.3389/fmedt.2021.640569)
Supplement: Supplementary file 2 [file Data_Sheet_2.docx]

Biomimetic LIFNano-CD4 for MS

METHODS continued: Measuring hLIF in the mouse brain LIF in mouse brain: ELISA usinghLIFNano-CD4 to spike brain homogenates followed by extraction and measurement.

**Method development (4)**

- **The following modifications to the standard ELISA were assessed to improve the LLOQ and hLIF extraction efficiency:**

| **COMPARISONS** | |
| --- | --- |
| Brain homogenised 1:4 w/v in **PBS** | Brain homogenised 1:4 w/v in **2.5% DMSO** |
| ELISA run with **200µl** sample | ELISA run with **50µl** samples |
| Incubation in ELISA plate for **24 hours** | Incubation in ELISA plate for **48 hours** |
| Incubation at **room temperature** | Incubation at **37°C** |

- **Levels of hLIFreleases from LIFNano particles (0.250 mg/ml) were assessed in these conditions.**

**Results (4)**

**
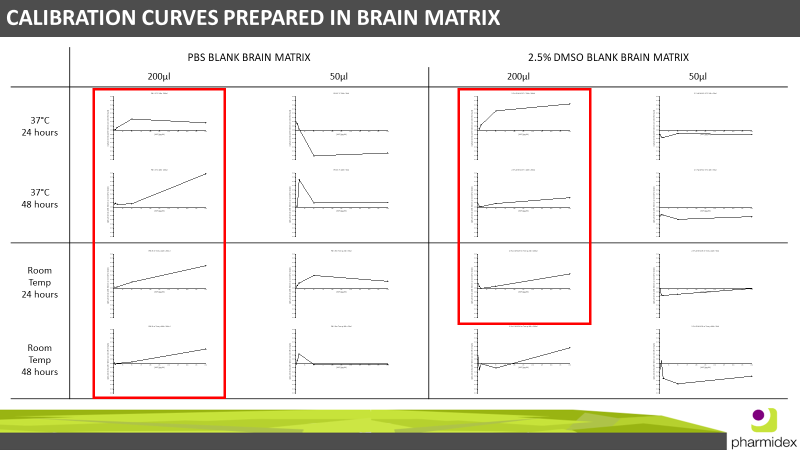
**

Figure 12 Effects of different parameters on hLIF quantification by ELISA

Figure 13Effects of different parameters on hLIF quantification by ELISA

**
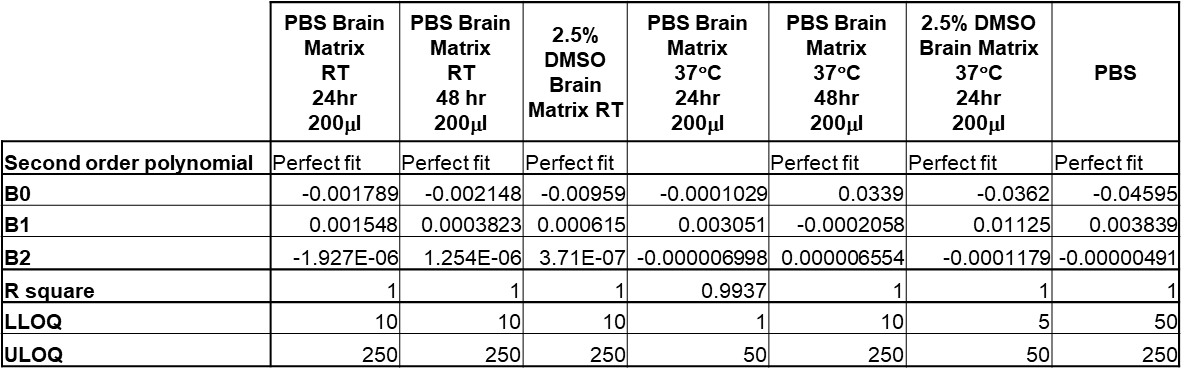
**

Figure 14 Extraction efficiency from LIFNano at RT under different conditions

Figure 15Extraction efficiency from LIFNano at 37 C under different conditions

**Conclusions (4)**

- Measuring hLIF using only 50µl of sample does not produce reliable standard curves.
- The data below were interpolated from standard curves shown in the previous slide. We did not analyse results for which we did not have reliable standard curves.
- hLIF levels were lower following incubation at 37°C compared to incubation at room temperature.
- hLIF levels were higher after 48 hours but the lower limit of quantification was higher than after a 24-hour incubation.
- Incubation of brain matrix homogenised in PBS for 24 hours appears to give the most reliable results.
- 48 hours incubation period may help to complete release of hLIF from NP
