# Supplementary figures and images for: Multiple Sclerosis: LIFNano-CD4 for Trojan Horse Delivery of the Neuro-Protective Biologic “LIF” Into the Brain: Preclinical Proof of Concept
Source: Front Med Technol. 2021 Apr 7;3:640569. doi: 10.3389/fmedt.2021.640569 (PMC8757767; doi:10.3389/fmedt.2021.640569)

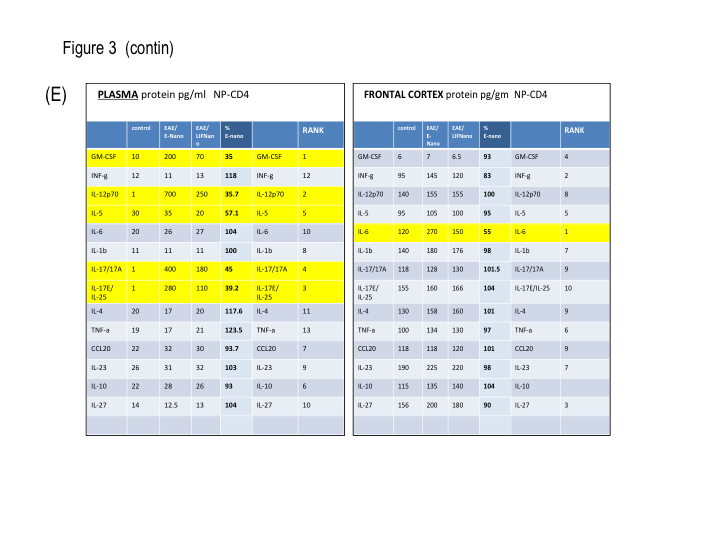

Supplement: Supplementary file 3 [file Image_1.tiff]
